# Supplementary material for: Adaptive Evolution Compensated for the Plasmid Fitness Costs Brought by Specific Genetic Conflicts
Source: Pathogens. 2023 Jan 13;12(1):137. doi: 10.3390/pathogens12010137 (PMC9861728; doi:10.3390/pathogens12010137)
Supplement: Supplementary file 1 [file pathogens-12-00137-s001.zip › Table S4-S6 Detailed functions of non-synonymous SNP-genes in Escherichia coli C600T, C600N500 and C600Y500.pdf]

pathogens

Supplementary Material

Adaptive Evolution Compensated the plasmid Fitness Costs brought by specific Genetic Conflicts

Feifeng Li [1†], Jiong Wang [1†], Ying Jiang [2], Yingyi Guo [1], Ningjing Liu [1], Shunian Xiao [1], Likang Yao [1], Jiahui Li [1], Chuyue Zhuo [1], Nanhao He [1], Baomo Liu [3*] and Chao Zhuo [1*]

Table S4. Detailed function of non-synonymous SNP-genes in E. coli C600 carrying the pHN330 plasmid.

Gene	Product and function	Supplementary description
ptrA	Protease III; hydrolyzes oligopeptides and peptides; involved in the protein catabolic process of bacteria.	Swamy et al. found that lack of PtrA has no effect on bacterial growth [1].
lpdA	Lipoamide dehydrogenase; component of the pyruvate dehydrogenase complex, which catalyzes the conversion of pyruvate to acetyl-CoA.	Murarka et al. found that pyruvate dehydrogenase deficient *E. coli* had decreased growth and glucose metabolism rates [2].
bamA	Outer membrane protein assembly factor BamA; component of the BAM complex, which is essential to constructing and maintaining the outer membrane.	Lack of BamA causes defects in assembly of β-barrel protein complex, influencing the construction of the outer membrane [3].
melA	α-Galactosidase; hydrolyzes melibiose to glucose and galactose.	Melibiose is a common nutrient. Belyaeva et al. found that mutants in the *melA* gene affect the growth of bacteria with melibiose as the carbon source [4].
gatA	Galactitol-specific PTS enzyme IIA component; involved in galactinol phosphorylation and transport.	Galactinol is transported into the cytosol by the PTS system and is a carbon source that provides energy for bacterial growth. Lengeler et al. found that bacteria with a mutated *gatA* gene cannot transport galactinol [5].
mdoB	Phosphoglycerol transferase I; catalyzes phosphoglycerol residues from phosphatidylglycerol into membrane-derived oligosaccharide.	Membrane-derived oligosaccharide plays an important role in maintaining the stability of the bacterial membrane [6].
yihV	6-Deoxy-6-sulfofructose kinase; involved in the sulphoglycolysis pathway.	A mutation in the *yihV* gene influences the utilization of sulfoquinovose in bacteria [7].
mntH	Divalent metal cation transporter MntH; transports most metal ions, with high affinity for Mn (II).	Makui et al. found that inactivation of MntH did not affect bacterial growth in nutrient rich medium [8].

trkA	NAD-binding component of Trk potassium transporters; K^+ uptake mediated by Trk transport system with hypotonic conditions.	TrkA plays an important role in maintaining potassium ion homeostasis under hypotonic conditions [9].
ydfK	Cold shock protein; unknown function.	It has been reported that after a cold shock, the expression of YdfK is upregulated [10].

Table S5. Detailed functions of non-synonymous SNP-genes in *Escherichia coli* C600N500.

Gene	Product and function	Supplementary description
bamA	Outer membrane protein assembly factor BamA; BamA is a component of the BAM complex, which is essential to constructing and maintaining the outer membrane.	Lack of BamA causes defects in the assembly of the β-barrel protein complex, influencing the construction of the outer membrane [3].
bcsG	Cellulose phosphoethanolamine transferase; the BcsE-BcsF-BcsG pathway controls the modification of cellulose by direct protein–protein interactions.	Exopolysaccharide cellulose is the major component of the self-produced extracellular matrix in biofilms, which enhances antibiotic resistance. The *bcsG* gene is essential for the formation of cellulose; lack of the *bcsG* gene results in the inability to form a biofilm [11].
lptF	Lipopolysaccharide transport system protein LptF; involved in lipopolysaccharide transport.	Sherman et al. found that LptF is essential for the transport of LPS. LPS plays a vital role in cell mobility, biofilm formation, antibiotic resistance, intestinal colonization, and is responsible for protecting the bacterial cell [12].
mreC	Cell shape determining protein MreC; *mreC* gene is a component of the *mreBCD* operon; encodes for cell cytoskeletal proteins.	The function of the bacterial cytoskeleton is highly conserved. Kruse T et al. found that the bacterial cell will undergo morphological change then lysis when the MreBCD protein is depleted [13].
sfmD	Fimbrial usher protein SfmD	Involved in the functioning of pili—macromolecular structures on the surface of gram-negative bacteria; defects in SfmD will not affect the biofilm formation of *E. coli* [14].

rfaF	ADP-heptose–LPS heptosyltransferase 2; involved in the synthesis of the inner core of LPS.	The inner core of LPS is highly conserved and responsible for the function of the outer membrane barrier [15].
gudD	D-glucarate dehydratase; involved in the D-glucarate catabolic pathway.	D-glucarate is not a common product in glucose metabolism [16].
araC	DNA-binding transcriptional dual regulator AraC; in the presence of arabinose, AraC stimulates the transcription of arabinose utilization genes.	Generally, bacteria utilize glucose as a carbon source due to carbon catabolite repression [17].
pepQ	Xaa-Pro dipeptidase; PepQ is capable of X-proline dipeptide hydrolysis.	Mutants in the *pepQ* gene of *E. coli* have no observable change in phenotype [18].
paaJ	Beta-ketoadipyl-CoA thiolase; the *paa* gene cluster is involved in phenylacetic acid metabolism.	Phenylacetic acid is not a common energy source.
dgt	Deoxyguanosinetriphosphate triphosphohydrolase; catalytic hydrolysis of dGTP to deoxyguanosine and triphosphate.	dGTPase is involved in maintaining proper dNTP pools and plays a vital role in cell survival and DNA replication. A mutation in the *dgt* gene reduces the fidelity of DNA replication [19].
mshA	D-inositol 3-phosphate glycosyltransferase; involved in the synthesis of extracellular colanic acid.	Colanic acid, an extracellular polysaccharide secreted by *E. coli*, is thought to promote biofilm formation and is important for survival. Ren et al. knocked out four consecutive genes of the CA operon (*wza*, *wzb*, *wzc*, and *wcaA*) and found that the strain was unable to synthesize colanic acid [20].

Table S6. Detailed functions of non-synonymous SNP-genes in *E. coli* C600Y500.

Gene	Product and Function	Supplementary description
fliY	Cystine ABC transporter periplasmic binding protein; FliY together with the L-cysteine exporter EAMA constitute the L-cysteine/L-cystine shuttle system involved in oxidative stress resistance	Ohtsu et al. found that deletion of the *fliY* gene results in decreased *E. coli* growth in LB broth [21].
bamA	Outer membrane protein assembly factor BamA; BamA is a component of the BAM complex, which is essential to constructing and maintaining the outer membrane.	Lack of BamA causes defects in the assembly of the β-barrel protein complex, influencing the construction of the outer membrane [3].
yghQ	Putative transport protein YghQ	No study of the *yghQ* gene has yet been conducted.

caiA	Crotonobetainyl-CoA reductase; Convert crotonobetainyl to γ-butyrobetaine under anaerobic conditions.	The expression of *caiA* suppressed is suppressed under aerobic conditions [22].
gloA	Glyoxalase I; involved in the glutathione-dependent glyoxalase I pathway that metabolizes methylglyoxal.	Endogenous synthesis of methylglyoxal occurs in the glycolytic pathway; 300 μM methylglyoxal causes growth arrest and higher concentrations cause cell death. The glutathione-dependent glyoxalase I pathway is the major pathway for methylglyoxal detoxification [23].
acnA	Aconitate hydratase A; catalyzes citrate and isocitrate via cisaconitate in the citric acid and glyoxylate cycles.	Mutations in the *acnA* gene has no effect on the growth of *E. coli* [24].
rlmB	23S rRNA methyltransferase RlmB; responsible for the methylation of Gm2251 in 23S rRNA	RlmB has no important role in ribosome assembly or function in *E. coli* [25].
ydiF	Acetate-CoA transferase; displays enzymatic activity with short-chain acyl-CoAs.	Rangarajan et al. found that YdiF is involved in metabolism of short-chain fatty acid [26].
sbp	Sulfate/thiosulfate ABC transporter periplasmic binding protein Sbp; regulates the composition of sulfate–thiosulfate transporters.	Mutants in the *sbp* gene do not affect thiosulfate and sulfate binding or uptake [27].
hycF	Formate hydrogenlyase subunit HycF; enables utilization of formic acid as a source of carbon in *E. coli*.	HycF functions only under anaerobic conditions [28].
tfaE	Putative tail fiber assembly protein TfaE.	No study of the *tfaE* gene has yet been conducted.
ycjX	Putative protein YcjX.	No study of the *ycjX* gene has yet been conducted.

References to the Supplementary Materials

1. Swamy, K.H.; Goldberg, A.L. Subcellular distribution of various proteases in Escherichia coli. *J Bacteriol* **1982**, *149*, 1027-1033, doi:10.1128/jb.149.3.1027-1033.1982.
2. Murarka, A.; Clomburg, J.M.; Moran, S.; Shanks, J.V.; Gonzalez, R. Metabolic analysis of wild-type Escherichia coli and a pyruvate dehydrogenase complex (PDHC)-deficient derivative reveals the role of PDHC in the fermentative

metabolism of glucose. *J Biol Chem* **2010**, *285*, 31548-31558, doi:10.1074/jbc.M110.121095.

3. Voulhoux, R.; Bos, M.P.; Geurtsen, J.; Mols, M.; Tommassen, J. Role of a highly conserved bacterial protein in outer membrane protein assembly. *Science* **2003**, *299*, 262-265, doi:10.1126/science.1078973.
4. Schmitt, R. Analysis of melibiose mutants deficient in alpha-galactosidase and thiomethylgalactoside permease II in Escherichia coli K-12. *J Bacteriol* **1968**, *96*, 462-471, doi:10.1128/jb.96.2.462-471.1968.
5. Lengeler, J. Mutations affecting transport of the hexitols D-mannitol, D-glucitol, and galactitol in Escherichia coli K-12: isolation and mapping. *J Bacteriol* **1975**, *124*, 26-38, doi:10.1128/jb.124.1.26-38.1975.
6. Fiedler, W.; Rotering, H. Properties of Escherichia coli mutants lacking membrane-derived oligosaccharides. *J Biol Chem* **1988**, *263*, 14684-14689.
7. Denger, K.; Weiss, M.; Felux, A.K.; Schneider, A.; Mayer, C.; Spiteller, D.; Huhn, T.; Cook, A.M.; Schleheck, D. Sulphoglycolysis in Escherichia coli K-12 closes a gap in the biogeochemical sulphur cycle. *Nature* **2014**, *507*, 114-117, doi:10.1038/nature12947.
8. Makui, H.; Roig, E.; Cole, S.T.; Helmann, J.D.; Gros, P.; Cellier, M.F. Identification of the Escherichia coli K-12 Nramp orthologue (MntH) as a selective divalent metal ion transporter. *Mol Microbiol* **2000**, *35*, 1065-1078, doi:10.1046/j.1365-2958.2000.01774.x.
9. Bossemeyer, D.; Borchard, A.; Dosch, D.C.; Helmer, G.C.; Epstein, W.; Booth, I.R.; Bakker, E.P. K+-transport protein TrkA of Escherichia coli is a peripheral membrane protein that requires other trk gene products for attachment to the cytoplasmic membrane. *J Biol Chem* **1989**, *264*, 16403-16410.
10. Polissi, A.; De Laurentis, W.; Zangrossi, S.; Briani, F.; Longhi, V.; Pesole, G.; Dehò, G. Changes in Escherichia coli transcriptome during acclimatization at low temperature. *Res Microbiol* **2003**, *154*, 573-580, doi:10.1016/s0923-2508(03)00167-0.
11. Thongsomboon, W.; Serra, D.O.; Possling, A.; Hadjineophytou, C.; Hengge, R.; Cegelski, L. Phosphoethanolamine cellulose: A naturally produced chemically modified cellulose. *Science* **2018**, *359*, 334-338, doi:10.1126/science.aao4096.
12. Sherman, D.J.; Xie, R.; Taylor, R.J.; George, A.H.; Okuda, S.; Foster, P.J.; Needleman, D.J.; Kahne, D. Lipopolysaccharide is transported to the cell surface by a membrane-to-membrane protein bridge. *Science* **2018**, *359*, 798-801, doi:10.1126/science.aar1886.
13. Kruse, T.; Bork-Jensen, J.; Gerdes, K. The morphogenetic MreBCD proteins of Escherichia coli form an essential membrane-bound complex. *Mol Microbiol* **2005**, *55*, 78-89, doi:10.1111/j.1365-2958.2004.04367.x.
14. Korea, C.G.; Badouraly, R.; Prevost, M.C.; Ghigo, J.M.; Beloin, C. Escherichia coli K-12 possesses multiple cryptic but functional chaperone-usher fimbriae with distinct surface specificities. *Environ Microbiol* **2010**, *12*, 1957-1977, doi:10.1111/j.1462-2920.2010.02202.x.
15. Cote, J.M.; Taylor, E.A. The Glycosyltransferases of LPS Core: A Review of Four Heptosyltransferase Enzymes in Context. *Int J Mol Sci* **2017**, *18*, doi:10.3390/ijms18112256.
16. Hubbard, B.K.; Koch, M.; Palmer, D.R.; Babbitt, P.C.; Gerlt, J.A. Evolution of enzymatic activities in the enolase superfamily: characterization of the (D)-glucarate/galactarate catabolic pathway in Escherichia coli. *Biochemistry* **1998**, *37*, 14369-14375, doi:10.1021/bi981124f.
17. Schleif, R. Regulation of the L-arabinose operon of Escherichia coli. *Trends Genet* **2000**, *16*, 559-565, doi:10.1016/s0168-9525(00)02153-3.
18. Miller, C.G.; Schwartz, G. Peptidase-deficient mutants of Escherichia coli. *J Bacteriol* **1978**, *135*, 603-611, doi:10.1128/jb.135.2.603-611.1978.
19. Gawel, D.; Hamilton, M.D.; Schaaper, R.M. A novel mutator of Escherichia coli carrying a defect in the dgt gene, encoding a dGTP triphosphohydrolase. *J Bacteriol* **2008**, *190*, 6931-6939, doi:10.1128/JB.00935-08.
20. Ren, G.; Wang, Z.; Li, Y.; Hu, X.; Wang, X. Effects of Lipopolysaccharide Core Sugar Deficiency on Colanic Acid

Biosynthesis in Escherichia coli. *J Bacteriol* **2016**, *198*, 1576-1584, doi:10.1128/JB.00094-16.

21. Ohtsu, I.; Wiriyathanawudhiwong, N.; Morigasaki, S.; Nakatani, T.; Kadokura, H.; Takagi, H. The L-cysteine/L-cystine shuttle system provides reducing equivalents to the periplasm in Escherichia coli. *J Biol Chem* **2010**, *285*, 17479-17487, doi:10.1074/jbc.M109.081356.
22. Elssner, T.; Hennig, L.; Frauendorf, H.; Haferburg, D.; Kleber, H.P. Isolation, identification, and synthesis of gamma-butyrobetainyl-CoA and crotonobetainyl-CoA, compounds involved in carnitine metabolism of E. coli. *Biochemistry* **2000**, *39*, 10761-10769, doi:10.1021/bi000776c.
23. MacLean, M.J.; Ness, L.S.; Ferguson, G.P.; Booth, I.R. The role of glyoxalase I in the detoxification of methylglyoxal and in the activation of the KefB K+ efflux system in Escherichia coli. *Mol Microbiol* **1998**, *27*, 563-571, doi:10.1046/j.1365-2958.1998.00701.x.
24. Varghese, S.; Tang, Y.; Imlay, J.A. Contrasting sensitivities of Escherichia coli aconitases A and B to oxidation and iron depletion. *J Bacteriol* **2003**, *185*, 221-230, doi:10.1128/JB.185.1.221-230.2003.
25. Lövgren, J.M.; Wikström, P.M. The rlmB gene is essential for formation of Gm2251 in 23S rRNA but not for ribosome maturation in Escherichia coli. *J Bacteriol* **2001**, *183*, 6957-6960, doi:10.1128/jb.183.23.6957-6960.2001.
26. Rangarajan, E.S.; Li, Y.; Ajamian, E.; Iannuzzi, P.; Kernaghan, S.D.; Fraser, M.E.; Cygler, M.; Matte, A. Crystallographic trapping of the glutamyl-CoA thioester intermediate of family I CoA transferases. *J Biol Chem* **2005**, *280*, 42919-42928, doi:10.1074/jbc.M510522200.
27. Sirko, A.; Zatyka, M.; Sadowy, E.; Hulanicka, D. Sulfate and thiosulfate transport in Escherichia coli K-12: evidence for a functional overlapping of sulfate- and thiosulfate-binding proteins. *J Bacteriol* **1995**, *177*, 4134-4136, doi:10.1128/jb.177.14.4134-4136.1995.
28. McDowall, J.S.; Murphy, B.J.; Haumann, M.; Palmer, T.; Armstrong, F.A.; Sargent, F. Bacterial formate hydrogenlyase complex. *P Natl Acad Sci USA* **2014**, *111*, E3948-E3956, doi:10.1073/pnas.1407927111.
